# Seroprevalence and Risk Factors of Brucellosis in Ruminants in Dhofar Province in Southern Oman

**DOI:** 10.1155/2022/3176147

**Published:** 2022-11-04

**Authors:** Waleed Al-Marzooqi, Elshafie I. Elshafie, AlGhalya Al-Toobi, Abeer Al-Hamrashdi, Kaadhia Al-Kharousi, Hatim El-Tahir, Maryne Jay, Yannick Corde, Yasmin ElTahir

**Affiliations:** ^1^Sultan Qaboos University, College of Agricultural & Marine Sciences, Department of Animal & Veterinary Sciences, P.O. Box 34, 123 Alkhod, Oman; ^2^Ministry of Agriculture & Fisheries, Directorate General of Animal Wealth, P.O. Box 567, 100 Muscat, Oman; ^3^Paris-Est University/Anses, EU/OIE/FAO & National Reference Laboratory for Brucellosis, Animal Health Laboratory, Maisons-Alfort, France

## Abstract

**Objective:**

The aim of the present work was to raise awareness of Brucella infection and emphasize the use of serological tests for screening and confirmation of the presence of the infection in different localities in the Dhofar region in the Sultanate of Oman.

**Methods:**

A seroprevalence of Brucella infection in naturally infected livestock was undertaken in 50 farms (a total of 434 sera, 207 goats, 84 sheep, 54 cattle, and 89 camels) from different wilayat of the Dhofar region in the southern part of Oman. Rose Bengal (RBT), complement fixation (CFT), and indirect enzyme-linked immunosorbent assay (I-ELISA) tests were used to determine the presence of Brucella antibodies. Statistical analysis (Pearson chi-square, binary logistic regression, and univariate logistic regression) was used to investigate the significance between the prevalence and the categorical risk factors individually, with two or more levels (animal species, animal condition, and or location).

**Results:**

Our results show that the overall seroprevalence based on CFT, RBT, and I-ELISA was 3% (13/424, CI: 1.8–5.1%), 4.8% (21/434, CI: 3.1–7.3%), and 8% (35/434, CI: 5.8–10.9%), respectively. The highest seroprevalence was reported in goats (13% (27/207)) and animals from East Jabal (13% (21/161)), whereas the lowest was recorded in camels (3.4% (3/89)) and animals from deserts (1.4% (1/69)). Parameters such as the positive predictive value (PPV) and the negative predictive value (NPV) showed that the sensitivity of I-ELISA and CFT based on the RBT test was 61.9% and 57.1%, respectively, whereas the specificity of I-ELISA (94.6%) was less than that of CFT (97.33%).

**Conclusion:**

We concluded that three tests are confirmatory for the presence of Brucella infection.

## 1. Introduction

A plethora of literature on brucellosis documented the worldwide occurrence of this zoonotic disease and the varying degrees of its presence in different countries [1–4]. As is the case in other countries, brucellosis was reported in both humans and animals in the Middle East, including the Arabian Gulf [5–9]. Virtually all *Brucella* infections in humans are acquired through direct contact with infected animals or consumption of infected animal products [4, 10, 11]. In animals, *Brucella* infection has a drastic impact on health and productivity, especially in farm animals, which constitute the source of staple food for many local communities, and also part of the global trade [12].

In Oman, brucellosis is entrenched in the Governorate of Dhofar, in the southern part of the country, where the pastoral and agro-pastoral communities depend for their livelihood on cattle, camels, goats, and sheep herding [13]. In Dhofar, brucellosis is classified as a public health problem, especially among the seminomadic Jabali population who live in close proximity to their livestock and traditionally consume raw camel's milk [13, 14]. *Brucella* seropositive ruminants in Dhofar region significantly exceeded those in the northern part of the country [15].

In Dhofar, goats, together with cattle and sheep, are natural hosts for *Brucella* [6]. Camels are not known to be primary hosts for any of the *Brucella* species, though they are susceptible to both *Brucella abortus* and *Brucella melitensis* [16]

Given the relatively humid climate in Dhofar during the monsoon season, there is more likelihood of survival of the *Brucella* organism and spread of infection. In addition, keeping a mixture of animals such as cattle, sheep, and goats, as is a common management practice in some areas, plays an important role in increasing the chances of cross-transmission of brucellosis. It is notable that the geographical location of Dhofar region in the route of animal trade traffic to Saudi Arabia makes it vulnerable to the potential threat from the influx of animals from endemic areas in East Africa and Yemen.

The objective of this study was an updated investigation on the seroprevalence of brucellosis and the risk factors associated with the infection in Dhofar region, the Sultanate of Oman, using blood samples from cattle, sheep, goats, and camels randomly selected. Providing such information on brucellosis in Dhofar region would assist in the strategic planning for brucellosis control and designing a sustained control program as well as applying urgent control measures.

## 2. Materials and Methods

### 2.1. Study Area, Design, and Sample Size

Considering resource constraints, 50 farms were selected randomly from three different districts of southern Oman (Dhofar, East Jabal and the desert region), namely, Salalah, Mirbat, Thumrait, Jibjat, Taqah, Madinat al Haqq, and Ghado from March through April 2015 (see map below). The study areas were selected based on the data on the animal population obtained from the Ministry of Agriculture and Fisheries [17] and also on information indicating possible Brucella infection in the selected areas.

**Figure 1:**
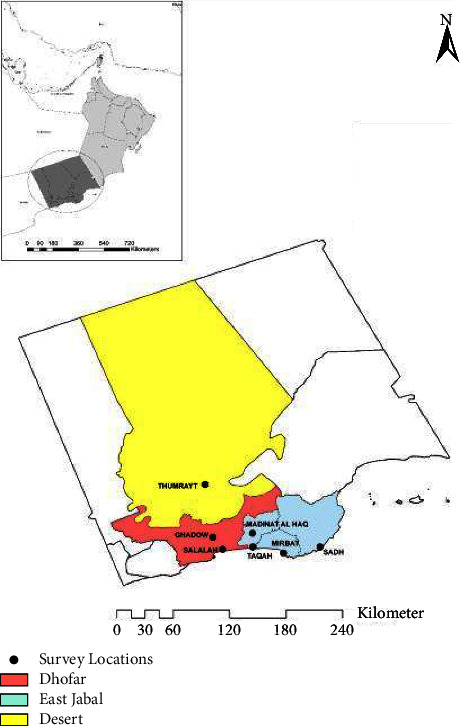
A map of Oman showing the three regions where the study was carried out.

Many farmers in the region have an aggressive attitude towards sampling their animals; therefore, we aimed to sample as many animals as we were allowed. Animals sampled were 434 in total, comprising 207 goats, 84 sheep, 54 cattle, and 89 camels. In the process of assessing animal condition based on the apparent health status of each animal, the overall sample size decreased where only 351 animals were left for further determination of risk factors. There was no record of a previous history of vaccination in the sampled areas. Most of the animals sampled were maintained under a mixed management system where different species were kept together except for camel herds (Figure1).

### 2.2. Ethical Consideration

Before blood sample collection, permission was granted by the Ministry of Agriculture and Fisheries to access farms in the study area. Collection of blood samples was carried out by professional veterinary technologists adhering to regulations and guidelines on animal husbandry. It was not an experimental research study on animals, and therefore, approval from the Ethics Committee of Sultan Qaboos University was not needed. The study did not involve endangered or protected species.

In each village, a meeting was held with community members to explain the purpose of the study. Farmers were not forced to donate blood from their animals. The name, region, and village of farmers were registered.

### 2.3. Blood Collection

Sampling was based on randomly selected herds and then randomly selected animals within those herds. The number of serum samples collected was 207 goats, 84 sheep, 54 cattle, and 89 camels, making a total of 434 samples. About 5 ml volume of blood was drawn from the jugular vein of each animal using a plain 10 ml vacutainer tube. Serum was separated by centrifugation, and the collected sera were stored at −20°C until analyzed.

### 2.4. Serological Tests

All serum samples were tested in the Brucellosis reference laboratory in Paris, France, according to the standards of the World Organization for Animal Health for diagnosis of brucellosis in small ruminants by using the Rose Bengal test (RBT) and the complement fixation test (CFT). The indirect enzyme-linked immunosorbent assay (I-ELISA) was carried out in the Department of Animal and Veterinary Sciences at Sultan Qaboos University.

### 2.5. Rose Bengal Test (RBT)

All serum samples were screened by the RBT (IDEXX batch 392–10) for the presence of antibodies against *Brucella* antigens. For the test, the positive and negative controls together with sample sera and the antigen were first equilibrated at room temperature. Equal volumes of 25 *μ*l of three replicates of the positive control, negative control, and sample sera were each mixed with 25 *μ*l of the antigen and then shaken in a rocker for four minutes. The degree of agglutination was then recorded as positive if there was visible agglutination or negative if there was no agglutination.

### 2.6. Complement Fixation Test (CFT)

The complement fixation test was carried out using IDEXX batch 79. The test and control sera were first inactivated for 30 min at 59°C. Using a 96-well round-bottomed microtitre plate, 25 *μ*l of each test and control serum was serially diluted from 1 : 2 to 1 : 128 in veronal buffer (VB). A volume of 25 *μ*l of *Brucella* antigen B115 was added to each well, followed by adding 25 *μ*l of guinea pig complement diluted 1 : 40. The plates were then incubated for 1 h in a water bath at 37°C. For the haemolytic system (HS), a mixture of equal volumes of hemolysin diluted 1 : 8 and sheep red blood cells diluted 1 : 30 was prepared and incubated at room temperature for 30 min. This was followed by dispensing 25 *μ*l of HS to each well and incubation of the plates again for 30 min at room temperature as prescribed [18, 19]. Positive and negative control sera were run on the same test plate in addition to the antigen control, complement control, and sensitized SRBC control. The plates were allowed to stand for 2 h at 4°C, and the reaction was observed visually. The end point was validated by observing complete haemolysis in control wells. The positive control serum was visualized at the expected titre + one dilution.

### 2.7. Enzyme-Linked Immunosorbent Assay (I-ELISA)

Samples tested with the Rose Bengal and CFT were further tested by the I-ELISA according to the manufacturer's instructions (IDEXX batch 4067). This diagnostic kit is designed to detect antibodies directed against *Brucella melitensis*, *Brucella abortus,* and *Brucella suis* in ovine, bovine, caprine, and porcine sera. In addition, it minimizes the cross-reaction with other Gram-negative bacteria.

The results of each test were compared with the results of other tests (RBT, I-ELISA, and CFT) as described in the Manual of the EU Reference Laboratory for Brucellosis (complement fixation test) [19].

### 2.8. Statistical Analysis

The prevalence of *Brucella* was calculated based on the following formula:

(1) $$\begin{array}{c} P\left(\%\right)=\frac{\text{Number of positive samples}}{\text{total number of animals}}\times 100. \end{array}$$

The univariate association between prevalence and categorical risk factors with two levels such as antibodies was individually assessed by testing its significance using the Pearson chi-square. Risk factors based on the antibody test and two categorical levels such as the location and animal species were investigated individually using univariate logistic regression. The model of binary logistic regression was performed (in the binary logistic regression model, we used backward stepwise methods) to test the significance of variables in the model and to test the significance shown by univariate analysis. The validity of the CFT and I-ELISA techniques was defined by using characteristic feature of sensitivity and specificity and parameters such as the positive predictive value positive (PPV) and the negative predictive value (NPV) based on RBT results. All statistical tests were conducted using SPSS version 20 (SPSS, IBM) at *α* = 0.05 significance level.

## 3. Results

### 3.1. Overall Seroprevalence of Brucella Infection in Different Ruminant Species

**Figure 2:**
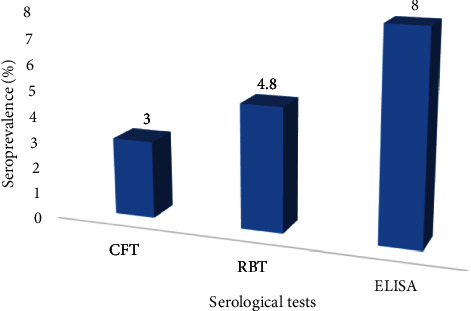
The overall seroprevalence of *Brucella* infection (%) in Dhofar using CFT, RBT, and I-ELISA.

As shown in Figure 2, the overall seroprevalence of Brucella infection in all ruminants in the three regions based on the CFT, RBT, and I-ELISA was 3% (13/424, CI: 1.8–5.1%), 4.8% (21/434, CI: 3.1–7.3%), and 8% (35/434, CI: 5.8–10.9%), respectively. It should be noted that 10 samples did not show reliable results in the CFT and were omitted, leaving the total number of samples to 424. The I-ELISA outperformed the CFT and RBT tests and was therefore used for further statistical analysis to correlate risk factors associated with the infection.

### 3.2. Univariate Analysis for the Association of the Risk Factors with *Brucella* Seroprevalence Using I-ELISA

To identify risk factors associated with *Brucella* infection, animal species and their location as well as their condition were subjected to univariate analysis using results obtained from the I-ELISA as it outperformed the CFT and RBT. As shown in Table 1, animal species and their location were factors which were significantly associated with *Brucella* seroprevalence (*p* < 0.05), whereas animal condition observed during sampling was not (*p* > 0.05). It should be noted that we were not able to observe the conditions for 83 animals, leaving the total number 351/434. The highest seroprevalence was reported in goats (13% (27/207)) and animals from East Jabal (13% (21/161)), whereas the lowest was recorded in camels (3.4% (3/89)) and animals from deserts (1.4% (1/69)). However, cattle in the desert location were not accessible.

#### 3.2.1. Binary Logistic Regression of Hypothesized Risk Factors Associated with Brucella Seroprevalence Using I-ELISA

As shown in Table 2, the likelihood of goats to be *Brucella* seropositive was about 4.3 and 4 times more likely than camels and sheep, respectively. The location of East Jabal was 10.2 and 2.2 times more likely to have circulating antibodies against *Brucella* than deserts and Dhofar, respectively.

#### 3.2.2. Comparative Analysis of the Sensitivity and Specificity of the Serological Tests

As shown in Tables 3 and 4, the result showed that the sensitivity of the I-ELISA and the CFT based on the RBT test was 61.9% and 57.1%, respectively. Therefore, we consider the I-ELISA to be more sensitive than the CFT in detection of *Brucella* seropositive cases, whereas the specificity of the I-ELISA (94.6%) was less than that of the CFT (97.33%); therefore, more caution should be exercised regarding a false positive result that may be observed by the I-ELISA.

## 4. Discussion

This study presents a recent report on such a high seroprevalence of *Brucella* infection in ruminants in Dhofar governorate, Sultanate of Oman. It is also the most recent study on the status of brucellosis in ruminants in the Dhofar regions, emphasizing that three serological tests (RBT, CFT, and I-ELISA) are important for screening and confirmation of presence of diseases [20]. Reports from the Ministry of Agriculture and Ministry of Health in Oman [17, 21, 22] and other reports [6, 14] confirm that the region is endemic for brucellosis and that almost all human and animal cases diagnosed in other parts of the country originated from Dhofar [8, 9]. Our results reflect the prevalence of Brucella infection in Dhofar region, mainly in sheep and goats but to a lesser extent in cattle and camels. This study confirms earlier serosurveillance reports which revealed the presence of *Brucella* antibodies in the sera of these animals in the region [6, 14–16]. It is important to mention that sera used in this study were mostly from privately owned farms. Our results are in agreement with reports [23] on higher prevalence of bovine brucellosis among privately owned animals than government farms; however, an earlier report [24] indicated otherwise.

In this study, the overall seroprevalence of *Brucella* infection in Dhofar region based on the CFT, RBT, and I-ELISA was 3% (13/424, CI: 1.8–5.1%), 4.8% (21/435, CI: 3.1–7.3%), and 8% (35/434, CI: 5.8–10.9%), respectively. In this study, 6/207 goat sera and 2/84 sheep sera tested positive in all three tests used. However, none of cattle or camel sera tested positive in all three tests. Moreover, 4/207 sera from goat sera tested positive in either I-ELISA/RBT (3) or RBT/CFT (1). On the other hand, none of sheep, cattle, or camel sera tested positive in two tests. 2/84 sheep sera tested positive in the I-ELISA (1) and RBT (1). This might be a result of cross-reacting antibodies produced due to infection with other Gram-negative bacteria. None of camel and cattle sera tested positive in the RBT or CFT. However, 3/89 (camel) and 2/54 (cattle) sera tested positive in the I-ELISA only. This could be explained by the following reasons: (i) higher sensitivity of the I-ELISA since it uses cytosolic S-LPS fragments, thus decreasing the cross-reaction with other Gram negative bacteria [8, 25–29], (ii) prozoning phenomena that occurs usually in acidified antigens in the RBT, and (iii) anticomplementary activity in the CFT [25]. As suggested by Bevins et al. [30], the presence of anti-*Brucellae* antibodies may not mean that animals have a current or active infection at the time of sample collection. These results therefore prompted us to define the validity of all three tests with regards to sensitivity and specificity.

The validity of the CFT and I-ELISA tests was defined by measurement of sensitivity and specificity and parameters such as the positive predictive value (PPV) and negative predictive value (NPV) based on RBT results as justified by an earlier report [20]. We showed that the sensitivity of the I-ELISA and the CFT based on the RBT test was 61.9% and 57.1%, respectively, whereas the specificity of the I-ELISA (94.6%) was less than that of the CFT (97.33%). These results are in agreement with our previous study [8, 31]. However, in comparison to both the CFT and RBT, the Bayesian method revealed that the I-ELISA had the best estimate [22]. Moreover, a report [32] concluded that the I-ELISA was a better serological test than both the RBT and STAT in terms of sensitivity, specificity, and rapidity and that it could be recommended for screening of brucellosis in sheep and goats. However, many reports recommended the use of a combination of two serological tests as confirmatory for the presence of *Brucella* antibodies in animals as all tests have limitations especially when screening individual animals [33, 34].

Our study correlated seroprevalence to potential risk factors including location, animal species, and animal condition, and though in the latter, 83 animals were excluded due to unapproachability as we could not actually closely approach animals and some owners were unwilling to cooperate according to their natural local attitude in such cases. As such, animal condition as a risk factor was presumed to be uncontrollable in that situation. Irrespective of animal condition, animal species, and location as well as risk factors, our results revealed a significant association with *Brucella* seropositivity (*p* < 0.05). The highest seroprevalence was reported in goats (13% (27/207)), whereas the lowest was recorded in camels (3.4% (3/89)). Moreover, binary logistic regression analysis revealed that the likelihood of goats to be *Brucella* seropositive was about 4.3 and 4 times more likely than camels and sheep, respectively. Our findings are in line with a report that seropositivity to *Brucella* infection was significantly higher in goats (5.8%) than in sheep (1.4%) [35]. The present results do not explain the preponderant prevalence of *Brucella* infection in goats in Dhofar, but it can be postulated that introduction of new and possibly infected animals for breeding may play a vital role in addition to mixing of animals, which is a risk factor for transmission of infection [36] which happens during grazing or frequently crowded market venues. Also, goats were suggested to be more susceptible to *Brucella* infection than sheep, and based on the observation that, unlike sheep, goats excrete the organism for longer periods which may increase exposure to organisms [37].

The highest seroprevalence was reported in goats (13% (27/207)) and animals from East Jabal (13% (21/161)), whereas the lowest was recorded in camels (3.4% (3/89)) and animals from deserts (1.4% (1/69)). The location of East Jabal was 10.2 and 2.2 times more likely to have circulating antibodies against *Brucella* than deserts and Dhofar, respectively. In agreement with our results, a study correlating seropositivity to location risk factors found that seroprevalence in livestock was significantly higher in lowlands than in highlands [38]. This pattern of distribution could not be precisely explained since many factors could be involved such as husbandry practice, seasonality, nomadism, and pastureland in different regions in the world. In Dhofar, southeastern monsoon, known as “khareef,” could influence animal management and so distribution of some species.

The present study indicates that animal movement between Oman and neighboring countries may have an impact on the transmission and spread of Brucella infection. Dhofar region has an active cross-border animal trade with Yemen which is active in trade with Ethiopia and Somalia where the disease is established, especially in their enormous population of small ruminants [38]. This has created an attractive market and influx of animals in cross-border trade through Dhofar and Yemen, legally and illegally, which warrants continuous monitoring of diseases, especially brucellosis, given the establishment of the disease there. This study was therefore conducted with the aim of charting an updated status of brucellosis in ruminant livestock in Dhofar by detecting *Brucella* antibodies among goats, sheep, cattle, and camels in the Dhofar region to outline measures for controlling brucellosis in that region.

## 5. Conclusion

Serological investigation revealed the highest prevalence of brucellosis in goats and to a much lesser extent in sheep, cattle, and camels in Dhofar in southern Oman. The use of the three tests RBT, CFT, and I-ELISA improved the detectability of seropositive animals, and the I-ELISA seemed to be of higher sensitivity than the RBT and CFT. Therefore, a combination of serological methods (RBT, CFT, and I-ELISA) can be used to detect the disease in domestic animals. Moreover, animal species and location risk factors revealed significant association with *Brucella* seropositivity. The highest seroprevalence was reported in goats (13% (27/207)) and animals (13% (21/161)) from East Jabal. We conclude that the three tests are important for detection of *Brucella* infection in the region.

Further studies are needed to identify the species of *Brucella* in the region and most importantly the true status of Brucella infection (acute versus chronic) by measuring IgG and IgM antibodies as acute cases may present with high, low, or undetectable concentrations of the antibody [39]. If acutely infected animals are detected, they can be culled, and transmission could be stopped from them.

## Figures and Tables

**Table 1:** Univariate analysis for the association of the risk factors with Brucella seroprevalence using the I-ELISA.

Risk factors	No.	I-ELISA		Chi-square	*p* value
		Positive (%)	Negative (%)		
Animal species					
Goat	207	27 (13)	180 (87)	13.24	0.004
Camel	89	3 (3.4)	86 (96.6)		
Cattle	54	2 (3.7)	52 (96.3)		
Sheep	84	3 (3.6)	81 (96.4)		
Total	434	35 (8.1)	399 (91.9)		

Location					
East Jabal	161	21 (13)	140 (87)	10.24	0.006
Desert	69	1 (1.4)	68 (98.6)		
Dhofar	204	13 (6.4)	191 (93.6)		
Total	434	35 (8.1)	399 (91.9)		

Animal condition					
Apparently diseased	29	3 (10.3)	26 (89.7)	0.090	0.76
Apparently healthy	322	28 (8.7)	294 (91.2)		
Total	351	31 (8.8)	320 (91.2)		

**Table 2:** Binary logistic regression of hypothesized risk factors associated with Brucella seroprevalence using the ELISA.

Risk factors	*β*	*SE β*	*p* value	Adjusted OR (95% CI)
Animal species				
Goat	0.00	—	—	1.000
Camel	−1.46	0.623	0.019	0.233 (0.069–0.788)
Cattle	−1.36	0.750	0.069	0.256 (0.059–1.114)
Sheep	−1.40	0.623	0.025	0.247 (0.073–0.837)

Location				
East Jabal	0.00	—	—	1.000
Desert	−2.32	1.034	0.025	0.098 (0.013–0.744)
Dhofar	−0.79	0.370	0.033	0.454 (0.220–0.937)

Note. *β*: logistic coefficients; SE: standard error; OR: odd ratio; CI: confidence interval.

**Table 3:** Sensitivity and specificity of the ELISA as shown by the RBT.

Test results	RBT			Sen	Sp	PPV	NPV
	Positive	Negative	Total				
**ELISA**							
**Positive**	13	22	35	61.90%	94.67%	37.14%	97.99%
**Negative**	8	391	399				
**Total**	21	413	424				

Sen: sensitivity; Sp : specificity; PPV: positive-predicted value; NPV = negative-predicted value.

**Table 4:** Sensitivity and specificity of the CFT as shown by the RBT.

Test result	RBT			Sen	Sp	PPV	NPV
	Positive	Negative	Total				
**CFT**							
**Positive**	12	1	13	57.14%	97.33%	92.30%	97.81%
**Negative**	9	402	411				
**Total**	21	413	424				

Sen: sensitivity; Sp : specificity; PPV: positive-predicted value; NPV = negative-predicted value.

## Data Availability

The datasets used and/or analyzed during the current study are available from the corresponding author on reasonable request.
